# Calorie Restriction Using High-Fat/Low-Carbohydrate Diet Suppresses Liver Fat Accumulation and Pancreatic Beta-Cell Dedifferentiation in Obese Diabetic Mice

**DOI:** 10.3390/nu16070995

**Published:** 2024-03-28

**Authors:** Xiao Lei, Emi Ishida, Satoshi Yoshino, Shunichi Matsumoto, Kazuhiko Horiguchi, Eijiro Yamada

**Affiliations:** Department of Internal Medicine, Division of Endocrinology and Metabolism, Graduate School of Medicine, Gunma University, Maebashi 371-8511, Gunma, Japan

**Keywords:** diabetes, dyslipidemia, obesity, *db*/*db* mice, calorie restriction, carbohydrate/fat ratio, pancreatic β cells, β-cell dedifferentiation, metabolic dysfunction associated steatotic liver disease, peroxisome proliferator-activated receptor

## Abstract

In diabetes, pancreatic β-cells gradually lose their ability to secrete insulin with disease progression. β-cell dysfunction is a contributing factor to diabetes severity. Recently, islet cell heterogeneity, exemplified by β-cell dedifferentiation and identified in diabetic animals, has attracted attention as an underlying molecular mechanism of β-cell dysfunction. Previously, we reported β-cell dedifferentiation suppression by calorie restriction, not by reducing hyperglycemia using hypoglycemic agents (including sodium-glucose cotransporter inhibitors), in an obese diabetic mice model (*db*/*db*). Here, to explore further mechanisms of the effects of food intake on β-cell function, *db*/*db* mice were fed either a high-carbohydrate/low-fat diet (db-HC) or a low-carbohydrate/high-fat diet (db-HF) using similar calorie restriction regimens. After one month of intervention, body weight reduced, and glucose intolerance improved to a similar extent in the db-HC and db-HF groups. However, β-cell dedifferentiation did not improve in the db-HC group, and β-cell mass compensatory increase occurred in this group. More prominent fat accumulation occurred in the db-HC group livers. The expression levels of genes related to lipid metabolism, mainly regulated by peroxisome proliferator-activated receptor α and γ, differed significantly between groups. In conclusion, the fat/carbohydrate ratio in food during calorie restriction in obese mice affected both liver lipid metabolism and β-cell dedifferentiation.

## 1. Introduction

According to the 10th edition of the International Diabetes Federation (IDF) Diabetes Atlas [1], the number of patients with diabetes, especially those with type 2 diabetes, has rapidly increased worldwide. By 2030, the number of patients with diabetes is expected to reach 648 million [1]. Lifestyle interventions can prevent or delay the onset of type 2 diabetes. Nutritional intervention can improve glycemic control, regardless of drug treatment [2,3]. It also has numerous benefits when considering the treatment of other metabolic diseases. It has also been reported that caloric restriction, especially fasting between meals, can prolong organismal longevity and delay aging, which were associated with improved glucose homeostasis, lower adiposity, and enhanced mitochondrial oxidation [4]. Although accumulating knowledge makes it possible to manage and prevent type 2 diabetes and its complications, the complete etiology and definitive cure for diabetes remains unclear.

In type 2 diabetes, basic treatments, such as diet, exercise, and oral hypoglycemic drugs, work well in the early stages. However, sometimes blood glucose levels gradually become dysregulated and may shift to an insulin-dependent state. This condition is called β-cell dysfunction, which is the core phenomenon in the progression of type 2 diabetes. The mechanism of β-cell dysfunction has not been fully unveiled, but some molecular mechanisms such as endoplasmic reticulum and oxidative stress, glucotoxicity and lipotoxicity, and increased apoptosis and increased inflammatory cytokines in β cells, have been proposed [5]. Notably, Talchai et al. identified the dedifferentiation of β cells in diabetic model mice, proposing this as a novel cause of β-cell dysfunction [6]. β-cell dedifferentiation also occurs in human diabetic islets [7]. As reported, β-cell dedifferentiation was prevented by insulin treatment in acquired β-cell dysfunction model mice [8] and by dietary restriction in obese diabetic model *db*/*db* mice [9]. Thus, it has attracted attention as a therapeutic target for β-cell dysfunction; however, the detailed mechanism of β-cell dedifferentiation has not yet been elucidated.

We previously showed that, in *db*/*db* mice, β-cell dedifferentiation was improved by dietary restriction and the administration of peroxisome proliferator-activated receptor (PPAR)γ agonists, but not by other hypoglycemic drugs that simply lowered the blood glucose levels [10]. This result indicates that both glucose metabolism, as well as systemic energy metabolism, including the intake, digestion, and absorption of nutrients (such as lipids) and nutrient metabolism in the liver and adipocytes, are involved in β-cell dedifferentiation in various ways. In this study, we focused on the liver, because the abnormality of energy metabolism in the organ, which is the key regulator of the processing and redistribution of ingested nutrients, is likely associated with β-cell function. Metabolic dysfunction-associated steatotic liver disease (MASLD) and diabetes are risk factors for each other [11,12]. In particular, MASLD is linked with insulin resistance [13,14]. The relationship between nutritional balance, MASLD, and type 2 diabetes has been studied [15,16,17]. For example, Feinman et al. argued that a carbohydrate-restricted diet is the first choice for treating diabetes [15]. Noakes et al. described that limiting carbohydrate intake improves insulin resistance, glycemic control, and MASLD in patients with type 2 diabetes [16]. However, according to some reports, a high-fat diet (HFD) for a short time exacerbates MASLD [17], and these findings remains controversial.

Regarding the prevention of β-cell dysfunction, a few studies have shown that dietary restriction improves β-cell function [18]. However, fewer studies are focusing on the “quality” of diet, i.e., the macronutrient balance within an equivalent calorie restriction. Therefore, in the current study, we focused on the “quality” of dietary restriction, hypothesizing that carbohydrate restriction better recovers β-cell dedifferentiation, in addition to fatty liver, when equivalent calorie restriction is implemented. The final aim of our study was to clarify the detailed molecular mechanism of MASLD and β-cell dedifferentiation, which may potentially be improved by a better-quality diet. This study aims to provide the molecular basis for a calorie restriction method for diabetes in clinical medicine and to elucidate the energy metabolism involved in β-cell dedifferentiation.

## 2. Materials and Methods

### 2.1. Animals

There was no alternative to animal experiments for achieving the objective of this study, which was to analyze the effects of dietary nutrients on pancreatic β-cell dedifferentiation. All animal experiments were performed in accordance with the rules and regulations of the Animal Care and Experimentation Committee in Gunma University (Permission number: 17-049, accessed on 31 October 2014).

BKS.Cg m +/+ -Lepr^db^/Jcl heterozygous mice (*db*/*+*) were purchased from CLEA Japan, Inc. (Kanagawa, Japan) and bred to homozygosity in order to obtain wild-type, *db*/*+*, and *db*/*db* mice. Three types of food with different essential nutrient balances were obtained from Research Diets (New Brunswick, NJ, USA). The mice were fed a normal diet (NM), which contained 17% fat and 59% carbohydrates; a high-fat low-carbohydrate diet (HF), which contained 43% fat and 33% carbohydrates; or a high-carbohydrate low-fat diet (HC), which contained 2.5% fat and 73.4% carbohydrates (Supplementary Table S1).

**Figure 1:**
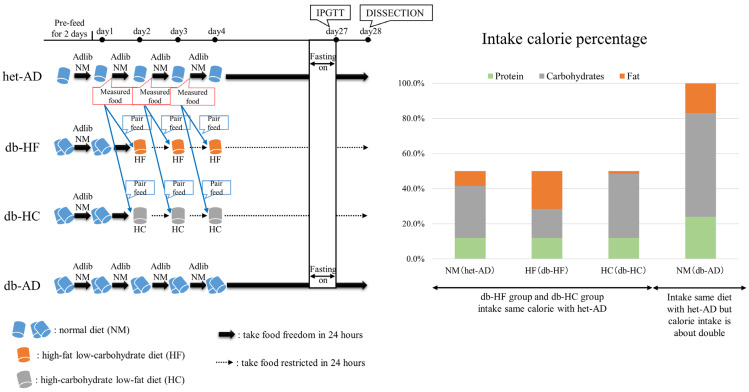
Study design for caloric restriction and nutrient balance modification in *db*/*db*. Left panel: Time schedule of mice feeding, IPGTT, and dissection. The pair-feeding method is also shown. Right panel: Percentage of major nutrients (protein, carbohydrate, and fat) consumed by mice in each group. The total calorie intake of the db-AD group was set as 100%. The Het-AD group took almost half of the calories when compared to the db-AD group. The ratio of carbohydrate/fat intake when fed HF and HC diets, assuming the same caloric intake as the het-AD group, is shown as HF (db-HF) and HC (db-HC).

Six-week-old male *db*/*db* mice were divided into three groups: db-HF, db-HC, and db-AD; *db*/*+* mice were assigned to the het-AD group. Before allocating the experimental groups, the body weight was measured and normalized. The food fed to mice were not blinded throughout the experiments. We fed the NM diet to the het-AD and db-AD groups, HF diet to the db-HF group, and HC diet to the db-HC group. The het-AD and db-AD groups had ad libitum access to food. The db-HF and db-HC groups were pair-fed with the het-AD group in order to restrict energy intake to a level comparable to that of the het-AD group (Figure 1). All animals were housed at 22 ± 1 °C temperature and 45 ± 5% humidity with a light/dark cycle (the light period was from 6:00 to 18:00 and dark period was from 18:00 to 6:00). The mice were allowed free access to water. All mice were fed an NM diet for 2 days before the experiment. During the experimental period (1 month), we measured the routine blood glucose level (using OneTouch Ultra Vue [LifeScan Japan, Tokyo, Japan]) and non-fasted body weight once a week. Each experiment was repeated at least three times. The experimental unit was a single animal.

### 2.2. Intraperitoneal Glucose Tolerance Test (IPGTT)

On the evening of the 28th day, after feeding the db-HF and db-HC groups, all mice were fasted. After 12 h, we measured the fasting blood glucose levels (on the morning of the 29th day). Subsequently, we injected 1.2 g/kg body weight of glucose intraperitoneally. After 15, 30, 60, and 120 min, the blood glucose levels were measured. After completing the IPGTT, pair-feeding was resumed until dissection.

### 2.3. Pancreatic Islets Isolation

Collagenase digestion was used to isolate the islets. Anesthetized mice were subjected to laparotomy, and 3 mg of collagenase P (Roche Diagnostics Deutschland GmbH, Mannheim, Germany) was injected into the bile duct. The expanded pancreas was dissected and heated at 37 °C for 15 min. Twenty milliliters of M199 Medium containing 10% fetal bovine serum (hereafter referred to as working medium) was placed in a tube, and the tissue was disrupted by shaking manually for approximately 30 s. The tissues were washed three times with the working medium. After washing, the tissues were passed through a φ500-µm metal filter to remove unbroken tissue pieces. The tissues were again washed twice. We then performed density gradient centrifugation using Histopaque-1077 (Sigma, St. Louis, MO, USA) to obtain an islet-rich fraction. The fractions were washed three times. Pellets containing a large number of pancreatic islets were resuspended in 10 mL RPMI Medium 1640 (Thermo Fisher Scientific, Paisley, UK). The islets were collected using a pipette under a stereomicroscope, some parts of the islets were centrifuged, and the pellets were frozen and stored at −80 °C for quantitative polymerase chain reaction (qPCR) analysis.

### 2.4. Glucose-Stimulated Insulin Secretion (GSIS)

The isolated islets were cultured overnight and preincubated with a buffer containing 2.8 mM glucose (the buffer contained 118.5 mM NaCl, 2.54 mM CaCl_2_, 1.19 mM KH_2_PO_4_, 1.19 mM MgSO_4_, 10 mM HEPES, 0.3 M BSA, pH 7.4) for 15 min. The buffer was then replaced with another buffer containing 2.8 mM or 16.7 mM glucose and incubated for 60 min. The supernatant was stored at −20 °C until the insulin concentration measurement. A Mouse Insulin Elisa kit (Mercodia, Uppsala, Sweden) was used to measure the insulin concentration in the supernatant.

### 2.5. Hematoxylin and Eosin (HE) Staining

The frozen sections were incubated at room temperature, washed with water for 2 to 3 min, and stained with hematoxylin (Muto Pure Chemicals Co., Ltd., Tokyo, Japan) for 8 min. After washing with water for 15 min, the sections were stained with eosin for 1 min. After washing with running water, the sections were dehydrated stepwise using various concentrations of ethanol and then incubated in xylene three times. The sections were sealed with Entellan New (Sigma, #107961), observed, and photographed under an optical microscope (OLYMPUS BX50 [Evident, Tokyo, Japan]).

### 2.6. Biochemical Examination of Blood and Liver

Skylight Biotech Co., Ltd. (Akita, Japan) was used for the biochemical analysis of the collected serum and liver tissues. Total cholesterol (T-CHO), low-density lipoprotein (LDL) cholesterol (LDL-CHO), high-density lipoprotein (HDL) cholesterol (HDL-CHO), and triglyceride (TG) concentrations were measured in the serum, and the T-CHO and TG content per 1 g of liver was measured for tissue lipid content.

### 2.7. qPCR

The RNeasy mini kit from QIAGEN (Dusseldorf, Germany) was used for RNA extraction, and the RNA extract was reverse transcribed using 0.1 mL of High-Capacity cDNA Reverse Transcription Kit (Applied Biosystems Inc., Foster City, CA, USA). We used Power SYBR™ Green PCR Master Mix (Applied Biosystems Inc.) for qPCR. Gene expression profiles were analyzed using primers targeting the gene sequences, as shown in Table S2. Since appropriate primers for SYBR were not available for some genes, a TaqMan Probe (Applied Biosystems Inc.) was used instead. To quantify the gene expression level, alpha-tubulin was used as an internal control, and the relative expression level was calculated using the 2^−ΔC_T_^ method. PCR was performed using an Applied Biosystems 7500 Sequence Detector with a standard of 40 cycles [19].

### 2.8. RNA Sequencing (RNA-Seq)

Liver RNA was extracted as described above and transported to DNAFORM (Yokohama, Japan) for sequencing. RNA-seq library preparation, sequencing, mapping, gene expression, and gene ontology (GO) enrichment analyses were performed using DNAFORM. The total RNA quality was assessed using a Bioanalyzer (Agilent, Santa Clara, CA, USA) to ensure that the RNA integrity number was >7.0. After poly (A) + RNA enrichment using the NEBNext Poly(A) mRNA Magnetic Isolation Module (New England BioLabs, Ipswich, MA, USA), double-stranded cDNA libraries (RNA-seq libraries) were prepared using SMARTer Stranded Total RNA Sample Prep Kit—HI Mammalian (Clontech, 634873, San Jose, CA, USA). RNA-seq libraries were sequenced using paired-end reads (50 nt for read 1 and 25 nt for read 2) on a HiSeqX instrument (Illumina, San Diego, CA, USA). The raw reads obtained were trimmed and quality-filtered using Trim Galore (version 0.4.4), Trimmomatic (version 0.36), and Cutadapt (version 1.16). The trimmed reads were then mapped to the mouse GRCm38.p6 (mm10) genome using the STAR software (version 2.7.2b). Reads on the annotated genes were counted using featureCounts (version 1.6.1). Fragments per kilobase of transcript per million (FPKM) mapped reads were calculated by normalizing them to total counts and transcripts. Differentially expressed genes (DEGs) were detected using the DESeq2 package (version 1.20.0). The list of DEGs detected by DESeq2 (base mean > 5 and fold-change < 0.25, or base mean > 5 and fold-change > 4) was used for GO enrichment analysis using the clusterProfiler package (3.16.0) [20]. We selected the top pathways using gene set enrichment analysis (https://www.gsea-msigdb.org/gsea/msigdb/index.jsp, accessed on 7 November 2023) and created a bubblemap using the R package (version 4.0.5) described above. Heatmap data were obtained by calculating z-scores from the raw FPKM data. For the raw data, we excluded genes if one of the two sample datasets in a group was less than five [21].

### 2.9. Fluorescent Immunostaining

The mice were anesthetized, and blood was collected from the heart chamber. We then performed perfusion fixation with 4% paraformaldehyde. The dissected pancreas and liver were processed into sliced frozen sections by the New Histo Science Laboratory Co. (Tokyo, Japan). For immunostaining, frozen sections were heated with Histo VT One (pH 7.0; NACALAI TESQUE, Inc. Kyoto, Japan) at 70 °C for 40 min. The sections were then blocked using Blocking One Histo (NACALAI TESQUE). Aldh1a3 (Novus Biologicals, Centennial, CO, USA, #NBP2-15339), insulin (BIO-RAD, Hercules, CA, USA, #5330-0104G), and glucagon (Sigma, #G2654-2ML) were utilized as primary antibodies, and the sections were incubated with these primary antibodies at 4 °C for 16 h. The following day, a secondary antibody (Invitrogen, Waltham, MA, USA, Alexa Fluor Plus 488, 568, and 647) was added, and the sections were incubated at room temperature for 1 h. After washing, the cells were stained with Hoechst 33342. Finally, the tissue area was covered with Prolong Gold Antifade reagent (Thermo Fisher Scientific, #P36930) and sealed with a cover glass. The sections were observed using a Zeiss LSM880 fluorescence microscope (Oberkochen, Germany). The number of positive cells (including strongly and weakly insulin-positive cells) was counted as previously described [10].

### 2.10. Statistical Analyses

We estimated the sample size using the following data; the expected fold change of the target gene expression estimated from RNA-seq analysis, and the standard deviation estimated from the previous studies in *db*/*db* mice. For estimation, α is set as 0.05 and β is set as 0.2. Data are reported as mean ± standard deviation. One-way analysis of variance (ANOVA) was performed for the comparison of means. When a significant difference was found, the Tukey–Kramer test was performed for post hoc analysis. Statistical significance was set at *p* < 0.05. Two-way ANOVA and the Wilcoxon rank-sum test were performed for the time-course study and for the comparison of medians, respectively. All statistical analyses were performed using JMP Pro14 (JMP, Cary, NC, USA).

## 3. Results

### 3.1. Calorie Restriction Lowered the Body Weight, Glucose Tolerance, and Insulin Secretion in Obese Mice, Regardless of the Carbohydrate/Fat Food Ratio

**Figure 2:**
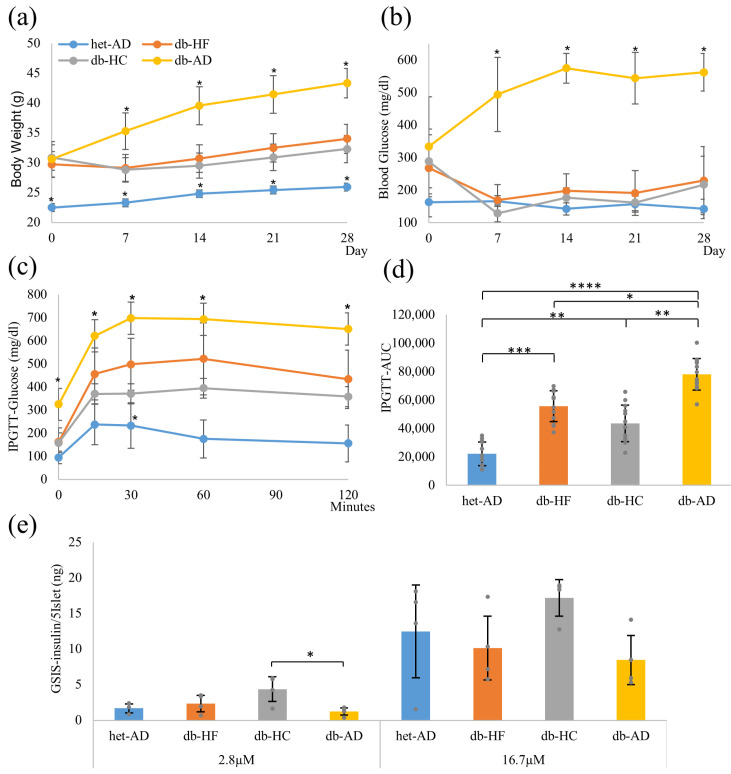
Basic glucose metabolism profiling. (**a**,**b**) Changes in body weight (**a**) and blood glucose levels (**b**) during diet intervention. (**c**) Serum glucose concentration during IPGTT. (**d**) AUC values of glucose concentration during IPGTT. (**e**) Insulin secretion during the GSIS analysis. Data are shown as means ± SD; (**a**–**d**) n = 12 and (**e**) n = 4 for each group. * *p* < 0.05 when compared to the db-HF group at (**a**–**c**). * *p* < 0.05, ** *p* < 0.01, *** *p* < 0.001, **** *p* < 0.0001 as compared to the het-AD group in (**d**). IPGTT, intraperitoneal glucose tolerance test; AUC, area under the curve; GSIS, glucose-stimulated insulin secretion; SD, standard deviation; HF, high-fat low-carbohydrate diet; HC, high-carbohydrate low-fat diet; het-AD, *db*/*+* mice fed normal diet ad libitum; db-AD, *db*/*db* mice fed normal diet ad libitum.

The db-HF and db-HC groups had a calorie equivalent to the energy intake of the het-AD group who were fed the NM diet ad libitum every 24 h. The db-AD group was fed the NM diet ad libitum. The body weight was significantly higher in the db-HF, db-HC, and db-AD groups than in the het-AD group; however, there were no significant differences among the three groups before calorie restriction (Figure 2a). Initially, there was no difference among the three db groups in blood glucose levels. After 4 weeks, the body weights of the db-HF and db-HC groups were significantly lower than those of the db-AD group, but were still significantly higher than those of the het-AD group. Blood glucose levels in the db-HF and db-HC groups were significantly lower than those in the db-AD group, but comparable to those in the het-AD group (Figure 2b).

IPGTT analysis and the area under the curve (AUC) of the blood glucose level change over time showed significant glucose intolerance in the db-HF and db-HC groups compared to that in the het-AD group (Figure 2c,d). In contrast, the db-HF and db-HC groups showed significantly improved glucose tolerance compared with that in the db-AD group. However, no significant differences were observed between the db-HF and db-HC groups. In the GSIS analysis, the db-AD group showed the lowest response; however, no significant difference was observed between the groups (Figure 2e). These results indicate that when calorie restriction was implemented in *db*/*db* mice to a level similar to that applied to the het-AD group, the glucose metabolism profile in individuals and body weight improved to the same degree regardless of the carbohydrate/fat ratio in food.

### 3.2. Calorie Restriction Using High-Carbohydrate Low-Fat Food Led to Hypercholesterolemia and Fat Accumulation in the Liver

**Figure 3:**
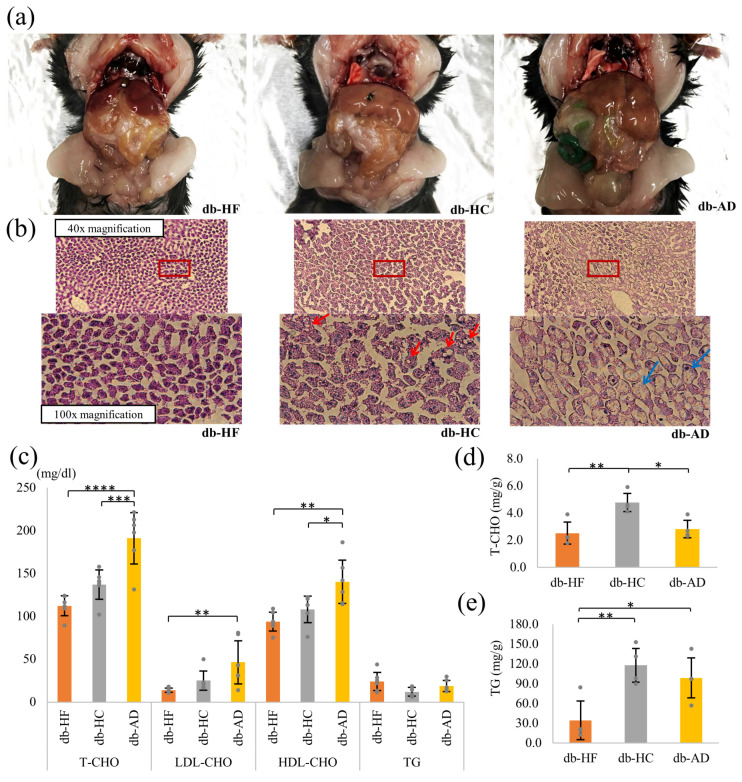
Fat accumulation in the liver. (**a**,**b**) Representative photograph of macroscopic (**a**) and microscopic HE stains (**b**) in the liver tissue of the db-HF, db-HC, and db-AD groups. (**b**) The upper photograph is 40× and the lower is 100× magnification. Red square in 40× photograph indicates the extent of the 100× photograph. Red arrow indicates macrovesicular droplet, and blue arrow indicates ballooning. (**c**) Serum concentration of lipid metabolites. (**d**,**e**) T-CHO and TG content per 1 g in the liver. Data are shown as means ± SD; (**c**) n = 6 and (**d**,**e**) n = 4 per group. * *p* < 0.05, ** *p* < 0.01, *** *p* < 0.001, **** *p* < 0.0001 as compared with indicated groups. HE, hematoxylin and eosin; T-CHO, total-cholesterol; TG, triglyceride; SD, standard deviation; HF, high-fat low-carbohydrate diet; HC, high-carbohydrate low-fat diet; AD, fed normal diet ad libitum.

Macroscopic findings at the time of dissection revealed that fat accumulation in the liver was more prominent in the db-HC group than in the db-HF group (Figure 3a). Similar findings were obtained for HE staining and Oil Red O staining of the liver (Figure 3b and Figure S1).

Serum T-CHO concentration was significantly lower in the energy-restricted groups than in the db-AD group (Figure 3c). The db-HF group had lower T-CHO levels than those in the db-HC group. The serum LDL-CHO concentration was significantly lower in the db-HF group than in the db-AD group. Serum HDL-CHO concentration showed a similar tendency to that of serum T-CHO level. In contrast, serum TG concentrations were lower in the db-HC group than in the db-HF group (Figure 3c). In addition, the T-CHO content in the liver tissue was significantly higher in the db-HC group than in the other groups (Figure 3d). The TG content of the liver tissues was significantly higher in the db-HC group than in the db-HF group (Figure 3e). Taken together, when calorie restriction was implemented in *db*/*db* mice, the low carbohydrate/fat ratio in food prevented the development of hypercholesterolemia and fat accumulation in the liver.

### 3.3. Calorie Restriction Using High-Carbohydrate Low-Fat Food Resulted in Increased Fatty Acid Elongation, Desaturation, and the Transportation of Long-Chain Fatty Acid in the Liver

**Figure 4:**
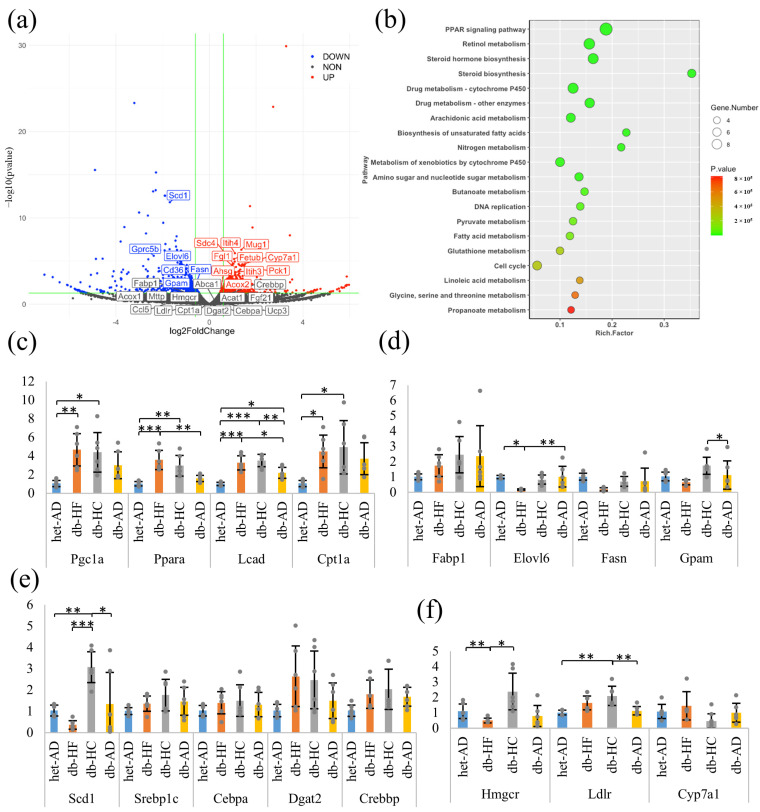
Transcriptome analysis in the liver. (**a**) Volcano map of DEG analysis in RNA-seq. Volcano plot displays the regulated transcripts in the db-HF group compared with those in the db-HC group. The vertical green lines correspond to two-fold up- and downregulation (log2 scaled), and horizontal green lines represent a *p* value of 0.05. The red points represent significantly upregulated genes in the db-HF group, and the blue points represent significantly downregulated genes in the db-HF group. (**b**) Bubble map of KEGG pathway enrichment analysis. Bubble plots exhibiting the top 20 significantly (*p* < 0.05) different genes enriched in the db-HF group compared with those in the db-HC group using GSEA. (**c**–**f**) The qPCR results of gene expression in the liver. Genes related to (**c**) fatty acid oxidation, (**d**) fatty acid metabolism, (**e**) fatty acid synthesis, and (**f**) cholesterol metabolism. Data are shown as means ± SD; n = 6 per group. * *p* < 0.05, ** *p* < 0.01, *** *p* < 0.001 as compared with indicated groups. DEG, differentially expressed gene; RNA-seq, RNA sequencing; KEGG, Kyoto Encyclopedia of Genes and Genomes; GSEA, gene set enrichment analysis; qPCR, quantitative polymerase chain reaction; SD, standard deviation; HF, high-fat low-carbohydrate diet; HC, high-carbohydrate low-fat diet; AD, fed normal diet ad libitum.

**Figure 5:**
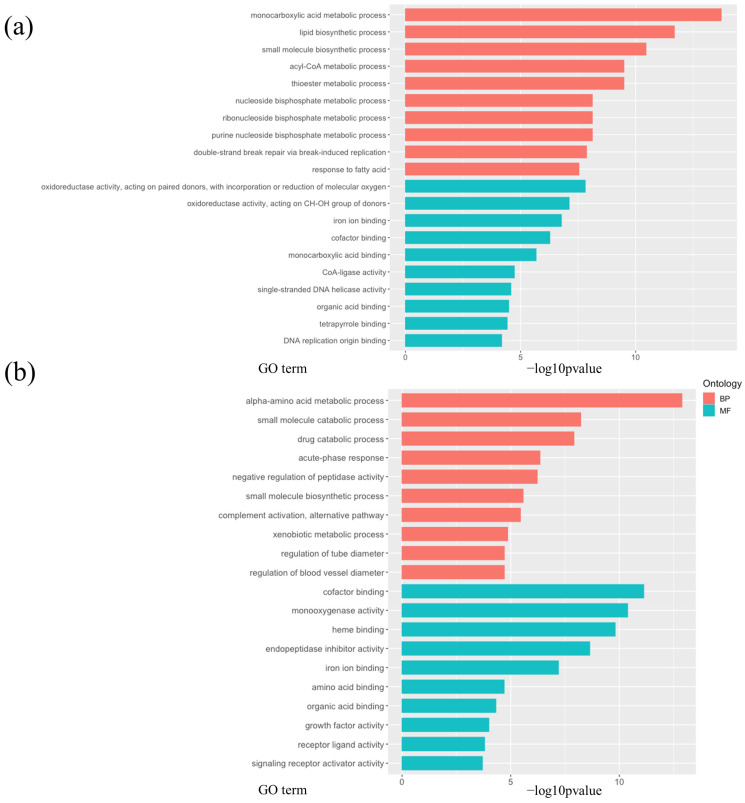
GO term enrichment analysis of the liver RNA-seq data. (**a**,**b**) The score of enriched GO terms in difference ontology. −log10 [*p* value] when comparing the db-HF and db-HC groups is shown (*p* < 0.05 is considered significant). (**a**) GO terms are more enriched in the db-HC group. (**b**) GO terms are more enriched in the db-HF group. The top 10 GO terms in each category are shown. All enriched GO terms are shown in Table S3. GO, Gene Ontology; RNA-seq, RNA sequencing; BP, biological process; MF, molecular function; HF, high-fat low-carbohydrate diet; HC, high-carbohydrate low-fat diet.

To investigate the molecular mechanism underlying the changes in lipid metabolism in the liver, the expression levels of genes involved in lipid metabolism were analyzed via RNA sequencing and qPCR analyses (Figure 3, Figure 4 and Figure 5). We focused on the energy-restricted groups to understand the differences between high-fat and high-carbohydrate nutritional balances. First, we comprehensively analyzed gene expression levels in the db-HF and db-HC groups using RNA sequencing and extracted DEGs using DEGSeq2. When compared to the db-HC group, there were 916 significantly DEGs, including 425 upregulated and 491 downregulated genes in the db-HF group (volcano map, Figure 4a). Next, we performed the Kyoto Encyclopedia of Genes and Genomes pathway enrichment analysis of the DEGs between the db-HF and db-HC groups to screen and classify the pathways (bulb map, Figure 4b). The top three pathways were the PPAR signaling pathway, retinol metabolism, and steroid hormone biosynthesis. In particular, genes such as *Fabp*, *Me1*, *Scd1*, and *Pltp* were downregulated, and *Cyp7a1* was upregulated in the PPAR signaling pathway in the db-HF group. Therefore, we hypothesized that lipid metabolism in the liver would be greatly affected when the carbohydrate/fat ratio in food differs in calorie restriction. RT-qPCR was performed to confirm the expression levels of each gene detected by the pathway analysis, and the results were similar to those obtained for RNA sequencing. There were no significant differences in fatty acid oxidation-related genes between the db-HF and db-HC groups (Figure 4c). PPARα was significantly increased in the energy-restricted group compared to that in the db-AD group. Regarding the fatty acid synthesis genes, *Scd1*, *Elovl6*, *Gpam*, and *Fabp1* showed significantly decreased expression in the db-HF group compared to those in the db-HC group (Figure 4d,e). Among the cholesterol metabolism-related genes, expression of *Hmgcr*, involved in cholesterol synthesis, was significantly elevated in the db-HC group compared to that in the db-HF group. Expression of *Cyp7a1*, involved in cholesterol excretion, tended to decrease in the db-HC group compared to that in the db-HF group (Figure 4f).

Thus, when calorie restriction was performed in a diet with a high carbohydrate/fat ratio, TG synthesis and accumulation in the liver increased, while TG levels in the serum decreased. It has been suggested that calorie restriction with HC food promotes an increase in cholesterol synthesis and a decrease in the excretion of bile acids; cholesterol is likely to accumulate in the liver.

**Figure 6:**
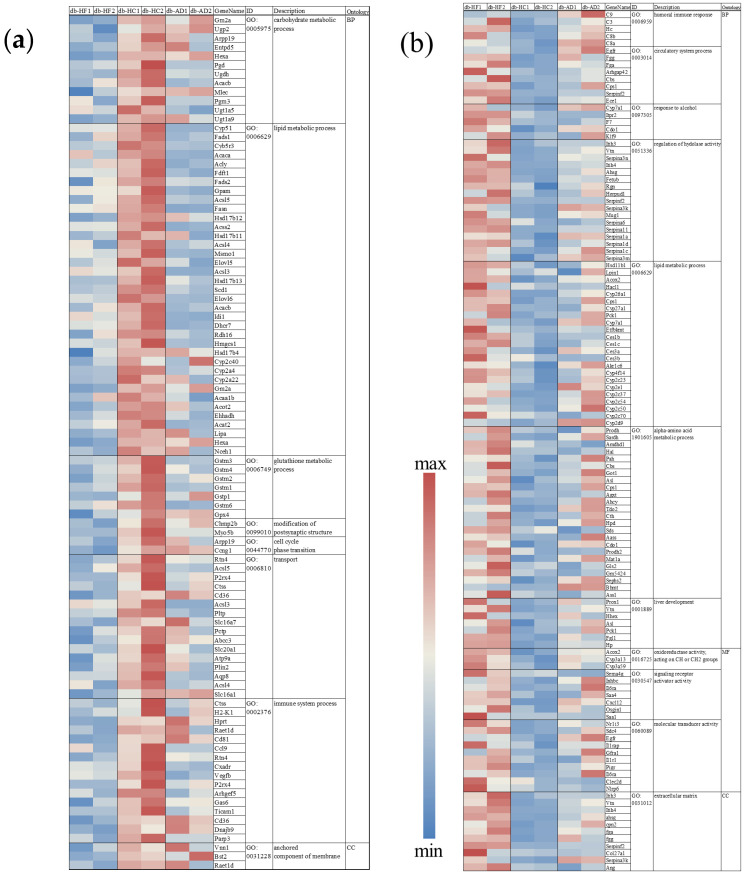
Heatmap for the genes included in the GO term enrichment analysis. (**a**,**b**) Heatmap showing the expression of each gene, with the respective GO term. The intensity increased from blue (relatively lower correlation) to red (relatively higher correlation). (**a**) Upregulated genes in the db-HF group. (**b**) Downregulated genes in the db-HF group. Original z-score data is shown in Figure S4. GO, gene ontology; BP, biological process; MF, molecular function; CC, cellular component; HF, high-fat low-carbohydrate diet; HC, high-carbohydrate low-fat diet; AD, fed normal diet ad libitum.

In addition, we performed GO enrichment analysis of the DEGs, finding that several biological processes other than lipid and carbohydrate metabolisms were affected in the livers of the db-HC and db-HF groups. Genes related to the cell cycle, such as *Ccng1*; glutathione reductase genes, such as *Gstm* and *Gstp1*; and immune regulation genes, such as *Cd36* and *Ccl* were all upregulated in the db-HC group (Figure 5a, Figure 6a and Figure S4a, and Table S3a). Genes encoding hydrolases, such as *Serpina*, *Itih*, and *Fetub*, and genes related to liver development, including *Pck1* and *Fgl1*, were significantly upregulated in the db-HF group (Figure 5b, Figure 6b and Figure S4b, and Table S3b).

### 3.4. Calorie Restriction Using Low-Carbohydrate High-Fat Food Better Restored the β-Cell Dedifferentiation Than High-Carbohydrate Low-Fat Food

**Figure 7:**
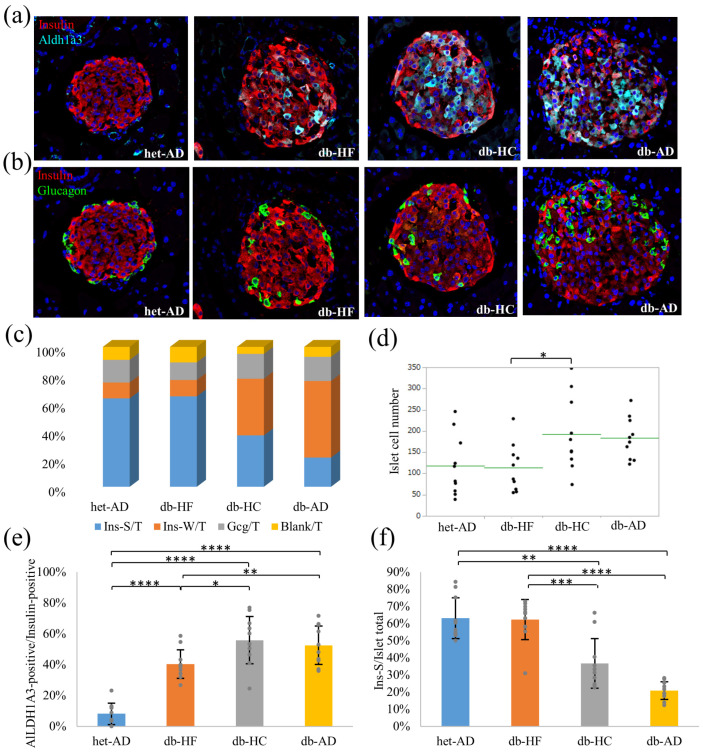
Immunofluorescent analysis in the pancreas. (**a**) Representative images of ALDH1A3/insulin co-immunostaining. (**b**) Representative images of glucagon/insulin co-immunostaining. blue, DAPI; red, insulin; cyan, aldh1a3; green, glucagon. (**c**) Percentage of positive cells in the islet. Ins-S and Ins-W indicate insulin-positivity with strong and weak fluorescent signals, respectively. Blank implies both insulin and glucagon-negative cells. (**d**) The cell number per islet section per group. (**e**) Number of ALDH1A3-positive cells among insulin-positive cells. (**f**) Number of Ins-S cells among the total islet cells. Data are shown as means ± SD; the number of mice is two per group; five islets are selected from each mouse. * *p* < 0.05, ** *p* < 0.01, *** *p* < 0.001, **** *p* < 0.0001 as compared with indicated groups. SD, standard deviation.

Immunostaining was performed to confirm the progress of β-cell dedifferentiation in the pancreatic islets (Figure 7). We chose ALDH1A3 as a β-cell dedifferentiation marker, as previously described [22]. In the db-HC group, more β-cells expressed ALDH1A3 than in the db-HF group (Figure 7a,e). The proportion of glucagon-positive cells was higher in the db-AD group (Figure 7b,c). The proportion of strongly stained insulin-positive cells was significantly higher in the db-HF group than in the db-HC group (Figure 7b,c,f). Despite the difference in the β-cell dedifferentiation between the db-HF and db-HC groups, there was no difference in glycemic control among individual animals, as shown in Figure 2.

## 4. Discussion

In the current study, we found that the development of fatty liver and the progression of β-cell dedifferentiation in *db*/*db* mice were inhibited more by an HF diet than an HC diet when the energy intake was reduced to that of *db*/*+*. This result suggests the correlation of β-cell dedifferentiation with hepatic steatosis and insulin resistance, which could be considered to be suppressed by carbohydrate restriction.

Dietary restriction controls the blood glucose levels in humans [23]. However, in our study, even though calorie restriction was implemented in *db*/*db* mice to the same level as that in *db*/*+* mice, glucose tolerance did not improve to the same level as in *db*/*+* mice. We restricted the diet for 1 month, but there was no difference in glycemic control via macronutrient balance between individuals in the db-HF and db-HC groups. There was no significant difference between energy restriction using HF and HC diets based on either the IPGTT or GSIS analysis results. In the GSIS analysis, we indicated the amount of insulin secreted from five islets and did not correct the amount using the number of β-cells per islet. In the db-HC group, the amount of insulin secreted before stimulation with glucose was higher than that in the other groups (Figure 2c), which suggests that the enlarged pancreatic islets in the db-HC might compensate for the insulin secretion ability in the individual animal (Figure 7d).

However, it was suggested that calorie restriction using the HC diet did not completely prevent the development of MASLD in *db*/*db* mice. One explanation is that excessive carbohydrate intake promotes TG synthesis and accumulation in the liver [24]. Serum TG levels were lower in the db-HC group than in the db-HF group, probably because of the low lipid intake from food. However, the TG content in the liver was the highest in the db-HC group (Figure 3e). In a study by Da Silva-Santi et al., carbohydrates promoted inflammation and facilitated the accumulation of neutral fats in the liver more than lipids [25].

According to the results of RNA-seq analysis in the liver tissue, there were several DEGs between the db-HF and db-HC groups, and these genes showed metabolic pathways affecting the two groups (Figure 3 and Figure 4). The main metabolic pathway responsible for these differences was the PPAR signaling pathway. In this pathway, four downregulated genes (*Pltp*, *Me1*, *Scd1*, and *Fabp*) and one upregulated gene (*Cyp7a1*) were identified as significantly DEGs (Figure S2). These changes in *Scd1* and *Cyp7a1* expression were consistent with the qPCR results.

PPAR has three isoforms, α, β, and γ. PPARs are members of the nuclear receptor superfamily and can be regulated by fatty acids [26]. The highest expression of the gene *Ppara* was found in the liver [27]. PPARα is a nuclear receptor involved in several metabolic pathways, such as fatty acid uptake and activation, intracellular fatty acid binding, mitochondrial and peroxisomal fatty acid oxidation, ketogenesis, TG turnover, lipid droplet biology, gluconeogenesis, and bile synthesis/secretion [28]. Long chain polyvalent unsaturated fatty acids, including fish oil and linolenic acid, readily bind to and activate PPARα [29,30,31]. There have been other reports of PPARα activation during HFD or fasting [32,33]. PPARγ is another member of the PPAR family, and one of its artificial agonists is thiazolidinedione. Thiazolidinedione improves glucose and insulin function in *ob*/*ob* mice [34,35]. Insulin sensitivity was increased in a PPARγ heterozygote knockout mouse on HFD [36]. Although PPARγ is mainly expressed in the adipose tissue [37], this report suggested that, in the absence of PPARα, PPARγ replaced the metabolic activity against HFD, similar to the role of PPARα in hepatic cells. In the current study, the hepatic *Ppara* and *Pparg* expression levels were not significantly different between the db-HF and db-HC groups. Nonetheless, expression of PPARα downstream genes, such as *Cyp4a10*, *Cyp4a14*, *Ehhadh*, *Aldh3a2*, *Fabp3*, *Fads2*, *Scd1*, *Cd36*, and *Lpl*, was lower in the db-HF group than in the db-HC group, suggesting that PPARα activity was lower in the db-HF group than in the db-HC group. Furthermore, the expression of genes involved in lipid synthesis, such as *Elovl6*, *Fasn*, and *Gpam*, was not significantly different according to qPCR analysis, but was significantly decreased in the RNA-seq analysis of the db-HF group. *Elovl6* and *Gpam* are additional downstream genes of PPARα [28]. Simultaneously, in the db-HF group, PPARγ activity was supposed to be decreased, as the downstream gene *Pepck*, which is linked with fatty acid oxidation and gluconeogenesis, was downregulated [38]. These findings in db-HF mice differ from those in HFD-fed mice in previous studies [37]. Body weight gain was observed in normal HFD-fed mice, but not in the db-HF group. In addition, leptin signaling was lacking in the livers of db-HF mice. The detailed mechanisms are unknown; however, the response of PPARα against a high fat ratio in food during calorie restriction is different from its response against calorie overload due to the high fat ratio in food. The decrease in overall PPARα activity alone cannot explain the metabolic changes in db-HF mice, as observed in PPARα-null mice, in which the liver was significantly fatty, serum glucose levels decreased, and ketone body increased [39]. In contrast, in db-HF mice, the fatty liver improved, and ketone body levels remained unchanged (Figure S3).

Fibrate, a PPARα agonist, activates PPARα, inhibits TG synthesis [40,41], and is used as an anti-hyperlipidemic drug. PPAR activators are also used for managing hepatic manifestations of MASLD [42]. Pemafibrate, a new member among the selective PPARα modulators, is commonly used in Japan to treat dyslipidemia [43,44]. Pemafibrate binds to PPARα and regulates the expression of PPAR-target genes, thereby decreasing serum TG concentration and increasing HDL-CHO concentration [45,46,47]. It also reduces liver TG levels in mice by stimulating hepatic PPARα to enhance fatty acid intake and β-oxidation [48]. Although pemafibrate did not significantly improve the liver fat content, as measured by magnetic resonance imaging-proton density fat-fraction, it significantly reduced liver stiffness, as measured by magnetic resonance elastography following long-term treatment in humans [49]. In mice, pemafibrate reversed steatohepatitis, and the expression of genes involved in inflammation (*Il6*, *Ccl5* and *Nlrp3*) and those related to cholesterol metabolism (*Lxra*, *Abca1*, *Mttp*, and *Cd36*) was decreased [50]. However, the genes related to energy metabolism and fat β-oxidation, such as *Ucp3*, *Cpt1a*, *Acox*, *Acat1*, and *Fgf21*, showed increased expression [43]. In the current study, the RNA-seq analysis of the liver tissue revealed a significant increase in *ll6* expression, but also highlighted a decrease in *Ccl5* expression in the db-HF group when compared to that in the db-HC group. Among the genes related to energy metabolism and β-oxidation, *Acox2* expression was significantly increased, and *Acox1*, *Ucp3*, *Cpt1a*, *Acat1*, and *Fgf21* expression also tended to increase in the db-HF group. The expression of CHO metabolism gene, *Cd36*, was significantly decreased in the db-HF group. Collectively, the pattern of hepatic gene modulation in db-HF mice was partly similar to that of the gene changes following treatment with pemafibrate, which does not simply activate PPARα, but fine-tunes the expression of downstream genes.

Among the genes not related to lipid metabolism, those related to cell cycle, glutathione reductase, and immune response were frequently upregulated in the db-HC livers, suggesting oxidative stress, inflammatory response, and high proliferation in hepatocytes. In contrast, serpinA family genes were more frequently detected in the db-HF livers in the GO enrichment analysis. This is consistent with the findings of a previous study that demonstrated that serpinA expression in the liver decreases as the disease progresses to NAFL/nonalcoholic steatohepatitis (NASH)/cirrhosis [51,52,53]. SerpinA knockout alters the expression of genes related to inflammation and lipid metabolism in the liver [51,54]. SerpinA is also associated with the prognosis of patients with hepatic cancer [54,55]. Based on the above evidence, serpinA is thought to be involved in different hepatosteatosis conditions in db-HF and db-HC mice.

In the present study, DEG analysis of the RNA-seq data showed that some genes, which were reported to be liver fibrosis/steatosis or liver injury markers, were upregulated in the db-HF group rather than in the db-HC group. Sdc4 [56,57] and Fetub [58], blood markers of NAFL in humans and those related to NASH and liver fibrosis were highly expressed in the db-HF group, despite their better fatty liver phenotype. Fetub expression is increased in patients with either diabetes or liver steatosis, and there is a positive correlation between its blood concentration and insulin resistance [58]. Furthermore, the expression of *Mug1*/*Itih3*,*4* [59,60]/*Fgl1* [61], which increases with liver damage in humans, was higher in the db-HF group than in the db-HC group. However, the other liver steatosis gene markers, such as *Thbs2*, *Lum*, *Lamc3*, *Lama2*, *Akr1b10*, *Col4a4*, *A2m*, and *C7* [62], were not significantly different between the db-HF and db-HC groups. Similarly, other liver inflammatory markers, such as *Cxcl9*, *Cxcl10*, and *Lyz* [63], also did not differ significantly. These discrepancies may be partly due to the differences in metabolism between mice and human livers. Further analysis is required to elucidate whether the livers of mice fed with a high-fat low-carbohydrate diet are more fibrotic, even though fat accumulation is lower than that in the livers of mice fed with a low-fat high-carbohydrate diet.

In summary, we hypothesize that, regardless of the lower total energy intake or weight loss, fat accumulation in the liver is one of the causes of fatty liver disease in mice on a high-carbohydrate diet.

Do et al. revealed that the islets were enlarged and insulin secretion increased in the early stages of diabetes in *db*/*db* mice [64]. Similar findings were observed in our study, more prominently in the db-HC group than in the db-HF group. Given that the proportion of strongly insulin-positive cells was significantly higher in the db-HF group than in the db-HC group, it was expected that more insulin would be secreted from each β cell in the db-HF group. In contrast, as the β cells in the db-HC group expressed more ALDH1A3 than the db-HF group, β-cell dedifferentiation progressed, and insulin secretion from each β cell was presumably reduced in the db-HC group; however, there was no significant difference between the db-HF and db-HC groups based on the GSIS results. As mentioned above, one possible cause is the compensation by the enlarged pancreatic islets in the db-HC group. In other words, a carbohydrate-restricted diet, such as the HF diet, could inhibit pancreatic β-cell dedifferentiation. Conversely, a high-carbohydrate diet, such as the HC diet, may induce β-cell dedifferentiation, possibly because of islet hypertrophy due to the metabolic compensation, which may be triggered by hepatic steatosis and exacerbated insulin resistance [65]. In humans, a low-carbohydrate diet, such as the Mediterranean diet [66], may improve β-cell function; however, the evidence is controversial [67]. Although β-cell dedifferentiation in the db-HC group was identified, an intervention study lasting longer than 1 month is needed to determine whether the β-cell mass could decrease in the db-HC group, similar to that in aged *db*/*db* or human patients with type 2 diabetes progression.

A report indicated that serpinB1 is secreted in the liver and regulates the pancreatic islet β-cell mass, thus improving islet function [68]. In our study, serpinA levels were increased in the livers of the db-HF group. Liver fat and islet dedifferentiation in the db-HF group were lower than those in the db-HC group. Although we did not measure serum serpinA and B1 levels, we cannot rule out the involvement of serpinA and serpinB secretions from the liver in improving islet dedifferentiation in db-HF mice. We hope to investigate this aspect in subsequent studies.

## 5. Conclusions

We show that carbohydrate restriction is more likely than fat restriction to improve fat accumulation in the liver and inhibit the progression of β-cell dedifferentiation in an obese mouse model when energy intake is restricted equivalently.

## Data Availability

The data presented in this study are available on request from the corresponding author.

## Supplementary Materials

The following supporting information can be downloaded at https://www.mdpi.com/article/10.3390/nu16070995/s1, Figure S1: Oil Red O staining in the liver [69]; Figure S2: Pathway map for the PPAR signaling pathway (KEGG # mmu03320) showing DEGs in the db-HF group; Figure S3: Serum ketone body and NEFA in the db-HF, db-HC, and db-AD groups; Figure S4: Original z-score data for heatmap in Figure 6; Table S1: Composition of NM, HF, and HC diets; Table S2: List of primer sequences used in SYBR & TaqMan-qPCR; Table S3: Complete GO enrichment analysis results.
