# Supplementary material for: Calorie Restriction Using High-Fat/Low-Carbohydrate Diet Suppresses Liver Fat Accumulation and Pancreatic Beta-Cell Dedifferentiation in Obese Diabetic Mice
Source: Nutrients. 2024 Mar 28;16(7):995. doi: 10.3390/nu16070995 (PMC11013071; doi:10.3390/nu16070995)
Supplement: Supplementary file 1 [file nutrients-16-00995-s001.zip › nutrients-2853978-supplementary.pdf]

Figure S1: Oil Red O staining in the liver.

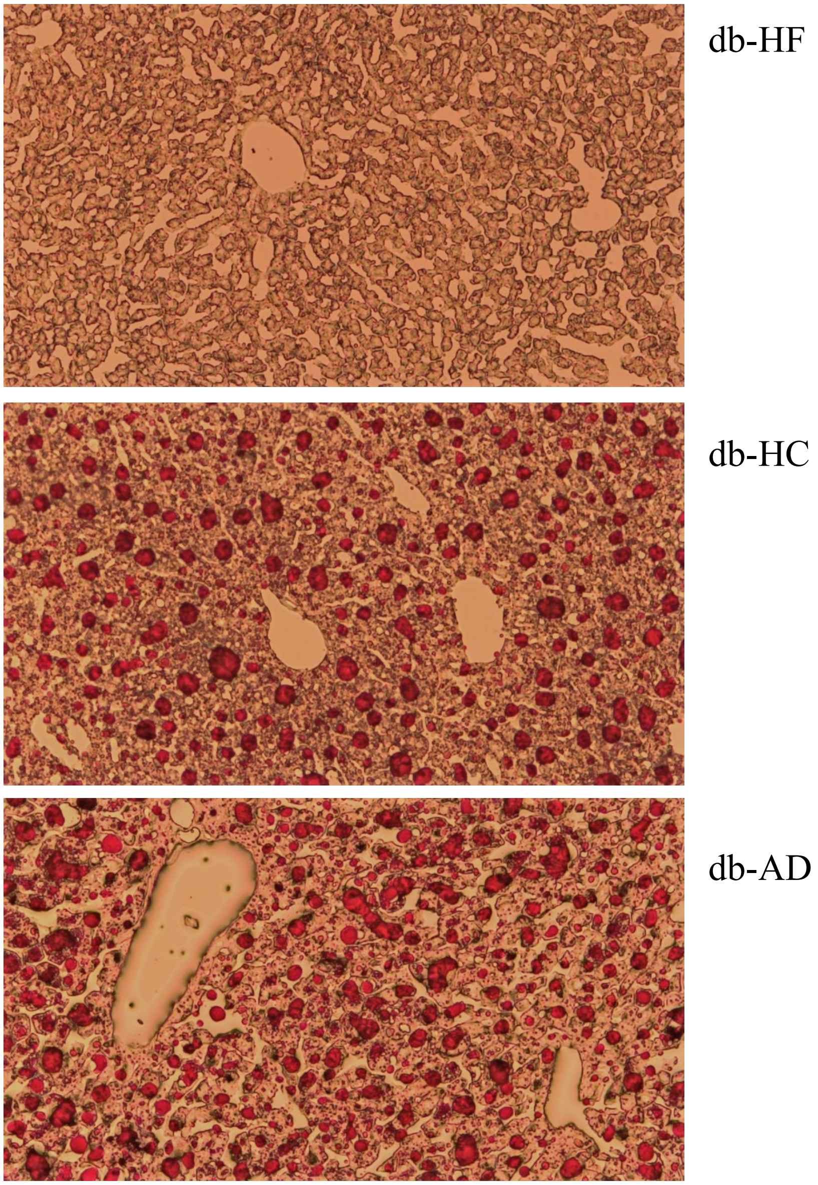

Oil Red O powder (Sigma, #O0625-25G) was dissolved in isopropanol at 60 °C for 30 min, and diluted with distilled water (ORO solution:dH2O 3:2) to make a working solution. After the frozen sections were returned to room temperature, the sections were washed with 60% isopropanol two times, and stained by Oil Red O working solution for 15 minutes. After washed with 60% isopropanol three times, the sections were washed with tap water for one minute. [69]

The sections were observed at 40x magnification with a LBD and ND filters, and photographed under an optical microscope (OLYMPUS BX50 [Evident, Tokyo, Japan]).

Figure S2: Pathway map for PPAR signaling pathway (KEGG # mmu03320) showing DEGs in the db-HF group.

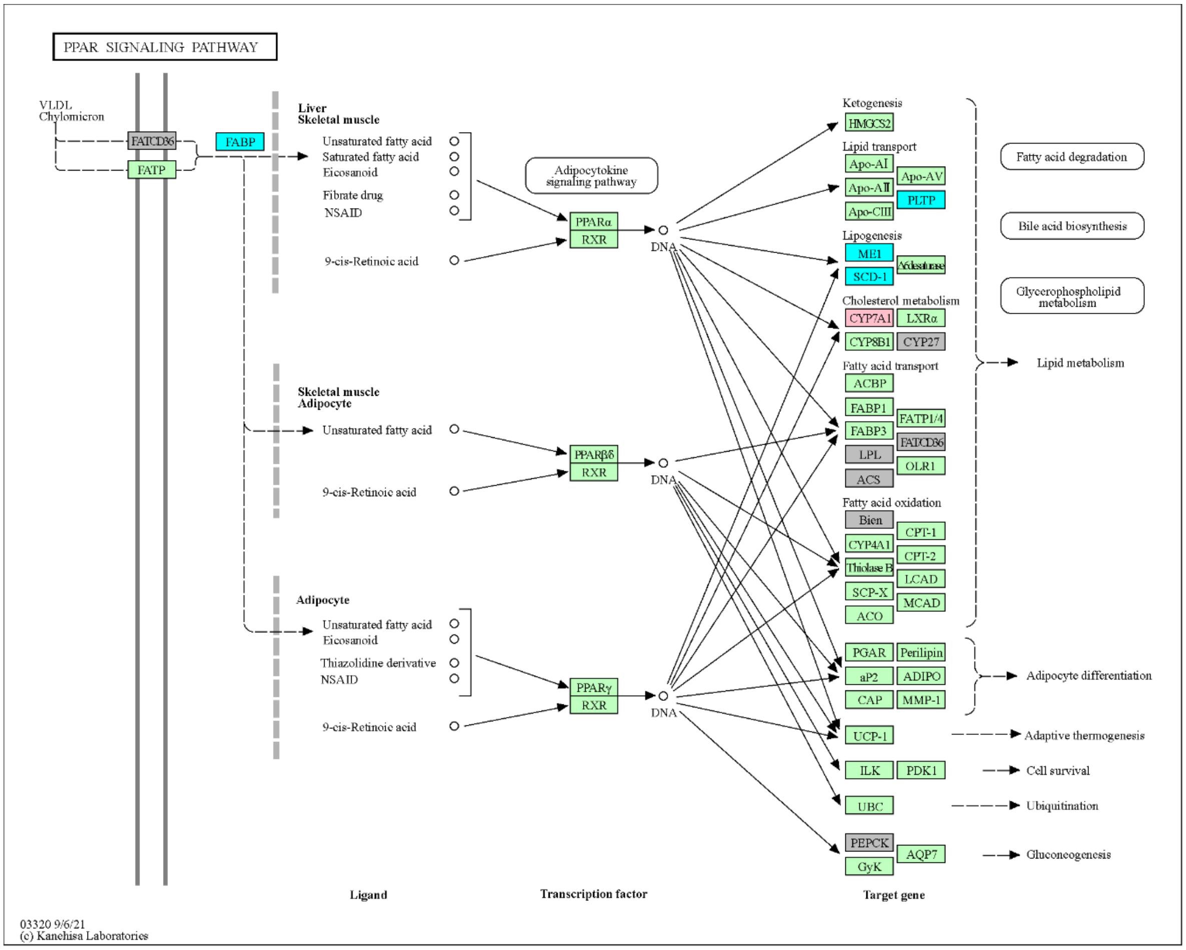

Genes highly expressed in the db-HF group compared to the db-HC group are highlighted in pink, while lower-expressed genes are marked in blue.

Figure S3: Serum ketone body and NEFA in the db-HF, db-HC and db-AD groups.

NEFA (uEq/L)

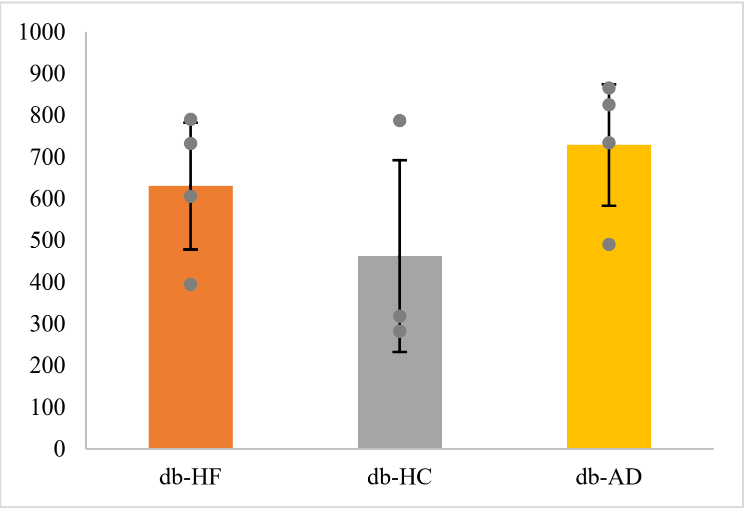

Total keton body (umol/L)

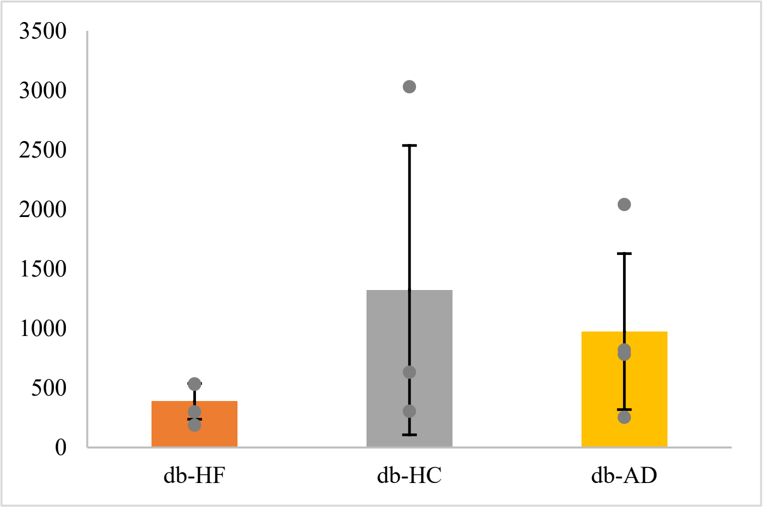

Data is shown as means ± S.D; n=3-4 for each group.

Figure S4: Original z-score data for heatmap in Figure 6.

S4a. List of more enriched DEGs in the db-HC compared to the db-HF group.

db-HF1	db-HF2	db-HC1	db-HC2	db-AD1	db-AD2	GeneName
-1.39	-1.19	0.64	1.05	-0.17	1.05	Gm2a
-0.62	-1.79	-0.14	0.86	0.50	1.19	Ugp2
-0.67	-1.33	0.33	1.67	0.67	-0.67	Arpp19
-1.23	-0.80	0.36	1.09	1.38	-0.80	Entpd5
-1.50	-1.25	0.75	0.75	0.25	1.00	Hexa
-0.72	-0.33	0.46	2.03	-0.72	-0.72	Pgd
-0.85	-1.01	1.01	1.69	-0.34	-0.51	Ugdh
-0.95	0.14	0.81	1.71	-0.99	-0.72	Acacb
-1.94	-0.59	0.23	0.68	0.68	0.95	Mlec
0.12	-1.28	0.46	1.86	-0.58	-0.58	Pgm3
0.23	-0.47	1.66	0.71	-0.78	-1.36	Ugt1a5
-1.38	-1.13	0.69	1.13	1.00	-0.31	Ugt1a9
-0.19	-0.16	0.81	1.71	-1.09	-1.09	Cyp51
-1.09	0.35	0.90	1.55	-1.00	-0.70	Fads1
-0.67	-0.49	1.55	1.25	-0.78	-0.86	Cyb5r3
0.55	-0.68	1.01	1.33	-1.07	-1.14	Acaca
-0.54	0.34	0.60	1.72	-1.20	-0.92	Acly
-0.16	-0.22	0.66	1.81	-0.88	-1.21	Fdft1
-1.67	0.10	0.93	1.42	-0.32	-0.46	Fads2
0.06	-0.01	0.27	1.87	-1.01	-1.18	Gpam
-1.17	0.05	0.85	1.70	-0.62	-0.80	Acsl5
-0.03	0.16	0.89	1.46	-1.27	-1.21	Fasn
-1.39	-1.09	1.00	1.19	0.60	-0.30	Hsd17b12
-0.19	-0.14	0.84	1.68	-1.17	-1.03	Acss2
-1.02	-0.58	1.44	0.68	0.73	-1.24	Hsd17b11
0.12	-1.36	0.86	1.60	-0.80	-0.43	Acsl4
-0.07	-0.36	0.64	1.83	-0.98	-1.07	Msmo1
-0.73	-0.23	1.67	0.90	-0.31	-1.31	Elovl5
0.27	-0.14	0.48	1.72	-1.17	-1.17	Acsl3
-0.86	-0.83	0.75	1.84	-0.14	-0.77	Hsd17b13
-0.77	-0.58	1.22	1.57	-0.53	-0.91	Scd1

-0.27	-0.50	1.60	1.11	-0.93	-1.01	Elovl6
-0.95	0.14	0.81	1.71	-0.99	-0.72	Acacb
0.31	-0.28	0.64	1.65	-1.12	-1.20	Idi1
-0.24	-0.11	0.80	1.71	-1.15	-1.02	Dhcr7
-1.36	0.20	1.20	1.29	-0.44	-0.90	Rdh16
-0.29	-0.46	0.57	1.95	-0.83	-0.95	Hmgcs1
-2.01	-0.22	0.74	0.72	0.91	-0.14	Hsd17b4
-1.23	-0.44	1.05	-0.12	-0.84	1.57	Cyp2c40
-0.88	-0.79	1.46	1.32	-0.79	-0.31	Cyp2a4
-0.92	-0.81	1.67	0.43	0.66	-1.03	Cyp2a22
-1.39	-1.19	0.64	1.05	-0.17	1.05	Gm2a
-0.93	0.53	1.42	0.78	-0.34	-1.45	Acaa1b
-1.33	-0.74	1.04	1.31	0.52	-0.80	Acot2
-0.98	-0.31	1.43	1.28	-0.34	-1.08	Ehhadh
-0.55	-0.02	0.90	1.69	-1.07	-0.94	Acat2
-1.20	-0.88	1.04	0.72	1.20	-0.88	Lipa
-1.50	-1.25	0.75	0.75	0.25	1.00	Hexa
-0.75	-1.17	1.39	0.53	0.96	-0.96	Nceh1
-0.62	-0.49	0.77	1.89	-0.84	-0.71	Gstm3
-1.18	-0.32	0.37	1.92	0.03	-0.83	Gstm4
-1.43	-0.47	0.69	1.69	-0.62	0.13	Gstm2
-0.77	-0.38	0.99	1.74	-0.86	-0.72	Gstm1
-1.03	-1.12	-0.15	1.51	-0.34	1.12	Gstp1
-0.56	0.17	1.05	1.49	-1.15	-1.00	Gstm6
-1.78	-0.56	-0.19	1.30	0.51	0.72	Gpx4
-0.87	-1.45	0.10	1.64	-0.10	0.67	Chmp2b
-0.97	-0.97	0.49	1.94	-0.24	-0.24	Myo5b
-0.67	-1.33	0.33	1.67	0.67	-0.67	Arpp19
-1.13	-1.56	0.14	0.99	0.99	0.57	Ccng1
-1.29	-0.75	0.31	1.91	0.04	-0.22	Rtn4
-1.17	0.05	0.85	1.70	-0.62	-0.80	Acsl5
-0.98	-0.98	0.20	1.96	-0.39	0.20	P2rx4
-0.59	-1.06	-0.12	2.01	-0.59	0.35	Ctss
-1.59	-1.04	0.46	0.31	1.36	0.51	Cd36
0.27	-0.14	0.48	1.72	-1.17	-1.17	Acsl3

-0.54	-0.36	1.25	1.50	-0.75	-1.10	Pltp
-0.88	-1.33	0.72	0.27	1.64	-0.42	Slc16a7
-1.53	-0.25	0.37	1.53	0.68	-0.80	Pctp
-1.67	-0.65	0.09	1.55	0.60	0.09	Abcc3
-0.48	-0.90	0.35	2.01	-0.07	-0.90	Slc20a1
-1.61	-0.23	0.32	1.70	0.32	-0.51	Atp9a
-1.45	-0.33	0.61	1.52	0.55	-0.90	Plin2
-0.44	-0.42	0.65	1.93	-0.74	-0.97	Aqp8
0.12	-1.36	0.86	1.60	-0.80	-0.43	Acsl4
-1.57	-1.00	-0.14	1.00	0.71	1.00	Slc16a1
-0.59	-1.06	-0.12	2.01	-0.59	0.35	Ctss
-0.94	-0.31	-0.24	1.96	-0.94	0.47	H2-K1
-1.22	-1.30	0.33	0.26	1.59	0.33	Hprt
-0.70	-1.30	0.90	0.70	1.30	-0.90	Raet1d
-1.36	-1.24	0.27	0.51	1.48	0.33	Cd81
0.27	-1.13	0.27	1.92	-0.67	-0.67	Ccl9
-1.29	-0.75	0.31	1.91	0.04	-0.22	Rtn4
-0.20	-0.43	0.35	2.03	-0.76	-0.98	Cxadr
-0.11	-0.34	0.80	1.72	-1.26	-0.80	Vegfb
-0.98	-0.98	0.20	1.96	-0.39	0.20	P2rx4
-0.27	-1.07	1.34	1.34	-1.07	-0.27	Arhgef5
-1.70	-0.50	0.55	1.60	-0.05	0.10	Gas6
-0.80	-0.80	0.57	1.94	-0.80	-0.11	Ticam1
-1.59	-1.04	0.46	0.31	1.36	0.51	Cd36
-1.13	-1.46	0.73	0.07	1.38	0.40	Dnajb9
-0.43	-1.30	0.87	1.73	-0.43	-0.43	Parp3
-1.55	-0.48	0.80	0.16	1.57	-0.48	Vnn1
-1.24	-1.02	0.56	0.34	-0.34	1.69	Bst2
-0.70	-1.30	0.90	0.70	1.30	-0.90	Raet1d

S3b. List of more enriched DEGs in the db-HF compared to the db-HC group.

db-HF1	db-HF2	db-HC1	db-HC2	db-AD1	db-AD2	GeneName
-0.62	-0.22	-0.89	-0.83	0.65	1.91	C9
-0.10	1.96	-1.21	-0.68	-0.31	0.34	C3
0.88	1.11	-1.41	-1.23	-0.04	0.70	Hc
1.53	-0.29	-1.09	-1.25	0.20	0.89	C8b
0.21	-0.28	-0.85	-1.49	1.03	1.37	C8a
-0.21	0.06	-0.91	-1.35	0.76	1.65	Egfr
0.85	0.99	-1.35	-1.14	1.02	-0.38	Fgg
0.47	1.67	-1.27	-1.13	0.34	-0.08	Fga
2.03	0.08	-0.65	-1.14	-0.41	0.08	Arhgap42
-0.02	2.06	-0.85	-0.95	-0.33	0.09	Cbs
0.90	0.78	-1.36	-0.93	-0.61	1.22	Cps1
1.42	1.33	-0.95	-0.99	-0.39	-0.43	Serpinf2
0.56	1.68	-1.05	-1.33	0.00	0.14	Ece1
0.99	-0.58	-0.97	-1.36	0.79	1.13	Cyp7a1
1.39	1.39	-0.28	-0.83	-0.83	-0.83	Itpr2
1.72	0.97	-0.67	-0.82	-0.97	-0.22	F7
1.20	0.24	-1.60	-1.07	0.56	0.67	Cdo1
-0.06	1.43	-1.00	-0.62	-1.00	1.25	Klf9
0.56	1.93	-1.16	-0.59	-0.35	-0.39	Itih3
0.50	1.71	-1.23	-1.08	-0.21	0.31	Vtn
1.85	0.12	-1.19	-1.05	0.25	0.02	Serpina3n
1.14	1.46	-1.14	-1.10	-0.27	-0.09	Itih4
1.29	1.04	-1.17	-1.23	-0.40	0.47	Ahsg
1.52	1.02	-1.00	-1.26	-0.06	-0.23	Fetub
0.97	0.94	-0.25	-1.94	-0.27	0.55	Rgn
-0.21	1.81	-0.53	-1.21	-0.62	0.75	Herpud1
1.42	1.33	-0.95	-0.99	-0.39	-0.43	Serpinf2
0.67	-0.45	-1.14	-1.28	1.22	0.97	Serpina3k
1.65	-0.12	-1.11	-1.28	0.34	0.53	Mug1
1.42	1.19	0.05	-1.13	-1.01	-0.53	Serpina6
1.22	1.43	-1.08	-1.08	-0.24	-0.24	Serpina11
0.45	0.60	-1.07	-1.69	0.92	0.79	Serpina1a
1.14	1.34	-0.57	-1.54	-0.46	0.09	Serpina1d
0.97	0.10	-1.17	-1.34	0.07	1.38	Serpina1c

0.81	-0.17	-1.75	-0.72	0.72	1.10	Serpina3m
1.31	1.09	-0.44	-1.42	-0.87	0.33	Hsd11b1
1.19	0.18	-0.70	0.00	-1.72	1.06	Lpin1
1.18	0.97	-1.52	-1.11	0.14	0.35	Acox2
2.19	-0.44	-0.03	-0.64	-0.64	-0.44	Hacl1
1.41	0.88	-1.24	-1.24	-0.18	0.35	Cyp26a1
0.90	0.78	-1.36	-0.93	-0.61	1.22	Cps1
0.90	1.50	-0.87	-0.95	-1.04	0.47	Cyp27a1
-0.21	1.56	-1.19	-0.77	-0.50	1.11	Pck1
0.99	-0.58	-0.97	-1.36	0.79	1.13	Cyp7a1
2.14	-0.09	-0.65	-0.93	-0.09	-0.37	Etfbkmt
1.61	1.10	-0.42	-1.19	-0.55	-0.55	Ces1b
1.07	1.52	-0.42	-1.26	-0.87	-0.04	Ces1c
1.41	-0.12	-0.93	-1.52	0.84	0.32	Ces3a
1.98	0.08	0.35	-0.74	-0.88	-0.79	Ces3b
0.48	0.34	-0.89	-1.72	0.52	1.28	Akr1c6
1.39	0.89	-0.60	-1.53	-0.60	0.46	Cyp4f14
1.42	0.62	-0.52	-1.53	-0.69	0.70	Cyp2c23
0.41	-0.04	-1.07	-1.42	1.53	0.59	Cyp2e1
0.46	0.87	-1.29	-1.25	-0.14	1.35	Cyp2c37
1.23	-0.38	-1.07	-1.20	0.10	1.32	Cyp2c54
0.22	-0.01	-0.98	-1.30	0.26	1.80	Cyp2c50
1.99	0.34	-0.08	-0.58	-1.15	-0.52	Cyp2c70
-0.17	-0.55	-1.19	-0.79	1.29	1.40	Cyp2d9
0.75	1.50	-0.43	-0.75	-1.50	0.43	Prodh
-0.26	1.32	-1.10	-1.25	0.12	1.17	Sardh
0.99	1.58	0.04	-0.67	-0.67	-1.27	Amdhd1
0.91	1.77	-0.58	-0.58	-1.08	-0.44	Hal
0.34	0.51	-0.76	-1.54	-0.17	1.62	Pah
-0.02	2.06	-0.85	-0.95	-0.33	0.09	Cbs
-0.04	0.88	-0.97	-1.26	-0.23	1.62	Got1
0.24	1.33	-1.69	-0.72	-0.06	0.90	Asl
0.90	0.78	-1.36	-0.93	-0.61	1.22	Cps1
0.97	1.74	-0.68	-0.90	-0.79	-0.35	Agxt
-0.34	1.41	-1.09	-1.00	-0.27	1.28	Ahcy
0.18	0.44	-1.41	-1.25	0.73	1.30	Tdo2
-0.27	1.66	-1.04	-0.82	-0.57	1.05	Cth

-0.28	1.14	-0.96	-1.41	0.23	1.28	Hpd
1.08	1.36	-0.40	0.20	-1.48	-0.76	Sds
0.42	0.77	-0.87	-0.97	-0.97	1.61	Aass
1.20	0.24	-1.60	-1.07	0.56	0.67	Cdo1
0.96	1.65	-0.01	-1.07	-0.84	-0.70	Prodh2
-0.16	0.99	-1.50	-1.00	0.42	1.25	Mat1a
0.19	2.03	-0.54	-0.21	-1.20	-0.27	Gls2
1.03	1.67	-0.32	-0.80	-1.07	-0.50	Gm5424
0.26	0.62	-1.62	-1.03	0.49	1.28	Sephs2
-0.33	-0.69	-0.90	-0.86	1.46	1.31	Bhmt
0.08	2.08	-0.25	-0.64	-1.07	-0.19	Ass1
1.79	-0.27	-1.15	-0.86	0.76	-0.27	Prox1
0.50	1.71	-1.23	-1.08	-0.21	0.31	Vtn
1.90	-0.32	-0.76	-0.91	0.71	-0.62	Hhex
0.24	1.33	-1.69	-0.72	-0.06	0.90	Asl
-0.21	1.56	-1.19	-0.77	-0.50	1.11	Pck1
1.12	1.20	-1.07	-1.31	0.50	-0.44	Fgl1
1.20	1.28	-1.33	-1.07	0.00	-0.09	Hp
1.18	0.97	-1.52	-1.11	0.14	0.35	Acox2
1.40	0.45	-1.03	-1.55	0.62	0.10	Cyp3a13
1.66	0.53	-0.69	-1.44	0.44	-0.50	Cyp3a59
1.83	0.71	-0.40	-0.16	-0.95	-1.03	Sema4g
-0.27	0.81	-0.99	-1.36	0.27	1.54	Inhbc
0.06	0.73	-1.13	-0.96	-0.45	1.75	Il6ra
0.70	1.18	-0.58	-1.32	-1.00	1.02	Saa4
0.74	0.74	-1.40	-1.40	0.89	0.43	Cxcl12
0.19	1.75	-0.58	-1.36	0.58	-0.58	Osgin1
2.22	-0.15	-0.50	-0.48	-0.52	-0.56	Saa1
1.70	0.52	-1.24	-1.05	0.33	-0.26	Nr1i3
0.76	1.41	-1.21	-1.04	-0.61	0.70	Sdc4
-0.21	0.06	-0.91	-1.35	0.76	1.65	Egfr
1.89	0.00	-0.38	-1.51	0.00	0.00	Il1rap
-0.64	1.04	-0.88	-1.00	-0.16	1.64	Gfra1
0.82	1.22	-0.41	-1.22	-1.22	0.82	Il1r1
0.82	1.52	-1.25	-1.09	-0.37	0.36	Pigr
0.06	0.73	-1.13	-0.96	-0.45	1.75	Il6ra
1.87	0.31	0.41	-0.86	-0.77	-0.95	Clec2d

2.10	0.17	-0.23	-0.87	-0.39	-0.79	Nlrp6
0.56	1.93	-1.16	-0.59	-0.35	-0.39	Itih3
0.50	1.71	-1.23	-1.08	-0.21	0.31	Vtn
1.14	1.46	-1.14	-1.10	-0.27	-0.09	Itih4
1.29	1.04	-1.17	-1.23	-0.40	0.47	ahsg
0.20	1.29	-1.37	-1.14	-0.04	1.06	cpn2
0.47	1.67	-1.27	-1.13	0.34	-0.08	fga
0.85	0.99	-1.35	-1.14	1.02	-0.38	fgg
1.42	1.33	-0.95	-0.99	-0.39	-0.43	Serpinf2
2.22	-0.25	-0.41	-0.58	-0.58	-0.41	Col27a1
0.67	-0.45	-1.14	-1.28	1.22	0.97	Serpina3k
0.97	1.67	-0.43	-0.58	-1.28	-0.35	Ang

Table S1: Composition of NM, HF, and HC diets.

**Product #**	**Normal Diet**		**High Fat Diet**		**High Carbo Diet**	
%	gm	kcal	gm	kcal	gm	kcal
Protein	24	***24***	28	***24***	22	***24.1***
Carbohydrate	59	***59***	38	***33***	68	***73.4***
Fat	8	***17***	22	***43***	1	***2.5***
Total		100		100		100
kcal/gm	4.0		4.7		3.7	
**Ingredient**	**gm**	**kcal**	**gm**	**kcal**	**gm**	**kcal**
Casein	240	960	240	960	240	960
L-Cystine	4	16	4	16	4	16
Corn Starch	387.5	1550	121	484	484	1936
Maltodextrin 10	100	400	100	400	150	600
Sucrose	100	400	100	400	100	400
Cellulose, BW200	50	0	50	0	50	0
Soybean Oil	76.5	689	65	585	11.4	103
Lard	0	0	130	1170	0	0
Mineral Mix S10026	10	0	10	0	10	0
DiCalcium Phosphate	13	0	13	0	13	0
Calcium Carbonate	5.5	0	5.5	0	5.5	0
Potassium Citrate, 1 H_2O	16.5	0	16.5	0	16.5	0
Vitamin Mix V10001	10	40	10	40	10	40
Choline Bitartrate	2	0	2	0	2	0
FD&C Yellow Dye #5	0	0	0	0	0.05	0
FD&C Red Dye #40	0	0	0.05	0	0	0
FD&C Blue Dye #1	0.05	0	0	0	0	0
**Total**	**1015.05**	**4055**	**867.05**	**4055**	**1096.45**	**4055**

Table S2: List of primer sequences used in SYBR & TaqMan-qPCR.

Gene Function	Gene Name	Gene Description	Base Sequence
**Relation of Fat Oxidation**	Pgc1a	peroxisome proliferative activated receptor, gamma, coactivator 1 alpha	F: TATGGAGTGACATAGAGTGTGCT
			R: GTCGCTACACCACTTCAATCC
	Ppara	peroxisome proliferator activated receptor alpha	F: AACATCGATTGTGAATATGTGG
			R: CCGAATAGTTCGCCGAAAGAA
	Lcad (Acadl)	acyl-Coenzyme A dehydrogenase, long-chain	F: TCTTTTCCTCGGAGCATGACA
			R: GACCTCTCTACTCACTTCTCCAG
	Cpt1a	carnitine palmitoyltransferase 1a	F: CTATGCGCTACTCGCTGAAGG
			R: GGCTTTCGACCCGAGAAGA
**Relation of Fat Synthesis**	Scd1	stearoyl-Coenzyme A desaturase 1	F: TTCTTGCGATACACTCTGGTGC
			R: CGGGATTGAATGTTCTTGTCGT
	Srebp1c (Srebf1)	sterol regulatory element binding transcription factor 1	F: ATCGGCGCGGAAGCTGTCGGGGTAGCGTC
			R: ACTGTCTTGGTTGTTGATGAGCTGGAGCAT
	Cebpa	CCAAT/enhancer binding protein (C/EBP), alpha	F: CCCAGCGGTGCCTTGTGC
			R: TCCTTCCCCCAGCCGTTAGTG
	Dgat2	ddiacylglycerol O-acyltransferase 2	F: TTCCTGGCATAAGGCCCTATT
			R: AGTCTATGGTGTCTCGGTTGAC
	Crebbp (Cbp)	CREB binding protein	F: GGCTTCTCCGCGAATGACAA
			R: GTTTGGACGCAGCATCTGGA
**Relation of Bile Acid Metabolism**	Hmgcr	3-hydroxy-3-methylglutaryl-co-enzyme A reductase	F: CTGGAATTATGAGTGCCCCAAA
			R: ACTCTATGGTGTCTCGGTTGAC
	Ldlr	low density lipoprotein receptor	Taqman : Mm00440169_m1
	Cyp7a1	cytochrome P450, family 7, subfamily a, polypeptide 1	Taqman : Mm00484152_m1
**Others**	Fabp1	fatty acid binding protein 1	F: ATGAACTTCTCCGGCAAGTACC
			R: CTGACACCCCCTTGATGTCC
	Elovl6	ELOVL family member 6, elongation of long chain fatty acids	F: GAAAAGCAGTTCAACGAGAACG
			R: AGATGCCGACCACCAAAGATA
	Fasn	fatty acid synthase	F: GGAGGTGGTGATAGCCGGTAT
			R: TGGGTAATCCATAGAGCCCAG
	Gpam (Gpat1)	glycerol-3-phosphate acyltransferase, mitochondrial	F: ACAGTTGGCACAATAGACGTTT
			R: CCTTCCATTTCAGTGTTGCAGA

Table S3: Complete GO enrichment analysis results.

S3a. List of GO terms more enriched in the db-HC compared to the db-HF group.

Pathway Description	Pvalue	Ontology
monocarboxylic acid metabolic process	1.92E-14	BP
lipid biosynthetic process	2.09E-12	BP
small molecule biosynthetic process	3.57E-11	BP
acyl-CoA metabolic process	3.24E-10	BP
thioester metabolic process	3.24E-10	BP
nucleoside bisphosphate metabolic process	7.66E-09	BP
ribonucleoside bisphosphate metabolic process	7.66E-09	BP
purine nucleoside bisphosphate metabolic process	7.66E-09	BP
double-strand break repair via break-induced replication	1.36E-08	BP
response to fatty acid	2.91E-08	BP
xenobiotic metabolic process	5.21E-08	BP
ribose phosphate metabolic process	2.74E-07	BP
fatty acid derivative metabolic process	1.15E-06	BP
organic hydroxy compound metabolic process	1.43E-06	BP
response to xenobiotic stimulus	2.82E-06	BP
DNA-dependent DNA replication	4.37E-06	BP
anion transport	5.62E-06	BP
response to lipoprotein particle	7.12E-06	BP
response to acid chemical	1.16E-05	BP
glutathione metabolic process	1.26E-05	BP
cellular response to lipoprotein particle stimulus	1.32E-05	BP
lipid catabolic process	2.28E-05	BP
response to bacterium	3.32E-05	BP
regulation of G2/M transition of mitotic cell cycle	8.46E-05	BP
drug transport	0.00010609	BP
macrophage cytokine production	0.00013514	BP
organic hydroxy compound biosynthetic process	0.00018484	BP
cellular hormone metabolic process	0.00019559	BP
myeloid leukocyte migration	0.00020903	BP
susceptibility to natural killer cell mediated cytotoxicity	0.00022562	BP
T cell mediated cytotoxicity	0.0002382	BP
response to stilbenoid	0.00025072	BP
positive regulation of myeloid leukocyte cytokine production involved in immune response	0.00025072	BP
cellular response to fatty acid	0.00028062	BP
cell cycle G2/M phase transition	0.00037903	BP
small molecule catabolic process	0.00051536	BP
regulation of protein kinase B signaling	0.00052314	BP
vitamin metabolic process	0.00052858	BP
carbohydrate metabolic process	0.00054329	BP
natural killer cell activation	0.0006727	BP

lipid localization	0.00077759	BP
import into cell	0.00087457	BP
DNA replication checkpoint	0.00096611	BP
lipoprotein transport	0.00096611	BP
lipoprotein localization	0.00096611	BP
inorganic ion homeostasis	0.0012638	BP
carboxylic acid transmembrane transport	0.0012964	BP
carbohydrate derivative catabolic process	0.00154089	BP
estrogen metabolic process	0.00166521	BP
cellular response to exogenous dsRNA	0.00166521	BP
inflammatory response	0.00169386	BP
amino sugar metabolic process	0.00184071	BP
production of molecular mediator of immune response	0.00196987	BP
positive regulation of ion transport	0.00211868	BP
positive regulation of leukocyte mediated cytotoxicity	0.00233388	BP
cellular response to acid chemical	0.00238944	BP
response to nutrient	0.00255313	BP
retinoic acid metabolic process	0.00265445	BP
defense response to virus	0.00291317	BP
syncytium formation	0.00296828	BP
positive regulation of lymphocyte mediated immunity	0.00298344	BP
cellular ion homeostasis	0.00314909	BP
mitotic DNA integrity checkpoint	0.00317276	BP
modified amino acid transport	0.00327406	BP
response to toxic substance	0.00354628	BP
response to reactive oxygen species	0.0036159	BP
flavonoid metabolic process	0.0038016	BP
positive regulation of response to external stimulus	0.00389088	BP
nitric oxide biosynthetic process	0.0046277	BP
lipid storage	0.0046277	BP
positive regulation of cell killing	0.0046277	BP
regulation of reactive oxygen species metabolic process	0.00466581	BP
response to interferon-alpha	0.00479411	BP
modification of postsynaptic structure	0.00479411	BP
antigen processing and presentation	0.00496743	BP
oxidoreductase activity, acting on paired donors, with incorporation or reduction of molecular oxygen	1.55E-08	MF
oxidoreductase activity, acting on CH-OH group of donors	7.74E-08	MF
iron ion binding	1.68E-07	MF
cofactor binding	5.36E-07	MF
monocarboxylic acid binding	2.12E-06	MF
CoA-ligase activity	1.86E-05	MF
single-stranded DNA helicase activity	2.64E-05	MF
organic acid binding	3.27E-05	MF
tetrapyrrole binding	3.81E-05	MF

DNA replication origin binding	6.66E-05	MF
transferase activity, transferring alkyl or aryl (other than methyl) groups	8.27E-05	MF
solute:sodium symporter activity	9.39E-05	MF
steroid dehydrogenase activity	0.00011482	MF
natural killer cell lectin-like receptor binding	0.00034764	MF
intramolecular oxidoreductase activity, transposing C=C bonds	0.00050764	MF
active ion transmembrane transporter activity	0.00050887	MF
sulfur compound binding	0.00059006	MF
symporter activity	0.00066941	MF
active transmembrane transporter activity	0.00068186	MF
DNA helicase activity	0.00076502	MF
modified amino acid binding	0.00081752	MF
oxidoreductase activity, acting on the CH-CH group of donors, NAD or NADP as acceptor	0.00106185	MF
glutathione binding	0.00129171	MF
oligopeptide binding	0.00129171	MF
DNA-dependent ATPase activity	0.00144348	MF
amide binding	0.00149045	MF
NADP binding	0.00265188	MF
C-acyltransferase activity	0.00267272	MF
vitamin binding	0.00347003	MF
steroid binding	0.00396085	MF
carboxylic ester hydrolase activity	0.00422604	MF
beta-2-microglobulin binding	0.00512492	MF
TAP binding	0.00512492	MF
glucose binding	0.00666402	MF
inorganic molecular entity transmembrane transporter activity	0.00758257	MF
oxidoreductase activity, acting on peroxide as acceptor	0.00760896	MF
T cell receptor binding	0.01048801	MF
cargo receptor activity	0.01203175	MF
MCM complex	5.13E-07	CC
anchored component of membrane	1.79E-05	CC
lipid droplet	1.98E-05	CC
brush border	2.24E-05	CC
brush border membrane	5.37E-05	CC
external side of plasma membrane	5.58E-05	CC
anchored component of plasma membrane	0.00030201	CC
MHC class I protein complex	0.00032605	CC
peroxisome	0.00034763	CC
microbody	0.00034763	CC
apical part of cell	0.00063442	CC
apical plasma membrane	0.0020591	CC
Golgi cisterna	0.00292146	CC

S3b. GO terms more enriched in the db-HF compared to the db-HC group.

Pathway Description	Pvalue	Ontology
alpha-amino acid metabolic process	1.32E-13	BP
small molecule catabolic process	6.02E-09	BP
drug catabolic process	1.23E-08	BP
acute-phase response	4.52E-07	BP
negative regulation of peptidase activity	6.02E-07	BP
small molecule biosynthetic process	2.69E-06	BP
complement activation, alternative pathway	3.59E-06	BP
xenobiotic metabolic process	1.39E-05	BP
regulation of tube diameter	1.97E-05	BP
regulation of blood vessel diameter	1.97E-05	BP
lipid catabolic process	3.08E-05	BP
fatty acid derivative metabolic process	3.41E-05	BP
regulation of actomyosin structure organization	3.91E-05	BP
hormone metabolic process	4.15E-05	BP
response to xenobiotic stimulus	5.75E-05	BP
cytolysis	5.78E-05	BP
liver development	6.41E-05	BP
urea cycle	6.74E-05	BP
fibrinolysis	7.68E-05	BP
negative regulation of hydrolase activity	0.00011581	BP
positive regulation of stress fiber assembly	0.00012055	BP
plasminogen activation	0.00012911	BP
vascular process in circulatory system	0.00014334	BP
nitrogen cycle metabolic process	0.00015283	BP
regulation of peptidase activity	0.00024898	BP
response to glucocorticoid	0.00037827	BP
negative regulation of multi-organism process	0.00039749	BP
dicarboxylic acid transport	0.00052629	BP
negative regulation of hemostasis	0.00068252	BP
cellular modified amino acid metabolic process	0.00068438	BP
negative regulation of coagulation	0.00077147	BP
bile acid biosynthetic process	0.00084055	BP
response to stilbenoid	0.00084055	BP
regulation of blood circulation	0.00086536	BP
organic hydroxy compound metabolic process	0.00107477	BP
sulfur compound catabolic process	0.00128016	BP
regulation of body fluid levels	0.00162951	BP
response to peptide hormone	0.00191938	BP
rhythmic process	0.00194724	BP
humoral immune response mediated by circulating immunoglobulin	0.00200377	BP
neutral lipid biosynthetic process	0.00200377	BP
acylglycerol biosynthetic process	0.00200377	BP
negative regulation of reproductive process	0.00200377	BP
thyroid hormone metabolic process	0.00210828	BP
regulation of mucus secretion	0.00210828	BP

polyol biosynthetic process	0.00223574	BP
regulation of ERK1 and ERK2 cascade	0.00271029	BP
heart trabecula formation	0.0027601	BP
response to alcohol	0.0032383	BP
cofactor binding	7.53E-12	MF
monooxygenase activity	4.16E-11	MF
heme binding	1.56E-10	MF
endopeptidase inhibitor activity	2.30E-09	MF
iron ion binding	6.35E-08	MF
amino acid binding	2.02E-05	MF
organic acid binding	4.83E-05	MF
growth factor activity	0.00010126	MF
receptor ligand activity	0.00015951	MF
signaling receptor activator activity	0.0002006	MF
vitamin binding	0.00033269	MF
oxidoreductase activity, acting on the CH-NH group of donors	0.00038162	MF
lyase activity	0.00043512	MF
steroid dehydrogenase activity, acting on the CH-OH group of donors, NAD or NADP as acceptor	0.00045898	MF
triglyceride lipase activity	0.00067293	MF
pyridoxal phosphate binding	0.00078159	MF
vitamin B6 binding	0.00088047	MF
oxidoreductase activity, acting on CH or CH2 groups	0.00105403	MF
steroid dehydrogenase activity	0.00137365	MF
extracellular matrix structural constituent	0.00139792	MF
heparin binding	0.00169367	MF
transforming growth factor beta receptor binding	0.00178769	MF
signaling receptor activity	0.0018659	MF
molecular transducer activity	0.0018659	MF
sulfur compound binding	0.00235597	MF
growth factor binding	0.0030583	MF
FAD binding	0.00678184	MF
protease binding	0.00801349	MF
glycosaminoglycan binding	0.00865039	MF
oxidoreductase activity, acting on single donors with incorporation of molecular oxygen, incorporation of two atoms of oxygen	0.00924161	MF
integrin binding	0.01011594	MF
transmembrane receptor protein kinase activity	0.01046474	MF
extracellular matrix	9.95E-05	CC
collagen-containing extracellular matrix	0.00010325	CC
